# The therapeutic effect of glucocorticoids on type II respiratory failure, heart failure, and massive pericardial effusion caused by hypothyroidism: A case report

**DOI:** 10.3389/fphar.2022.900701

**Published:** 2022-10-17

**Authors:** Jingyue Wang, Xiangjun Li, Botao Shen, Shipeng Wang, Jiahuan He, Yushi Wang

**Affiliations:** Department of Cardiology, The First Hospital of Jilin University, Changchun, China

**Keywords:** hypothyroidism, heart failure, glucocorticoids, type II respiratory failure, dyspnea, invasive ventilator assisted ventilation, severe case report

## Abstract

**Background:** Hypothyroidism is a disease commonly observed in outpatient clinics but can occasionally cause severe cardiovascular and respiratory diseases requiring hospitalization.

**Case report:** The patient reported herein suffered from heart failure, massive pericardial effusion, type II respiratory failure, and hypothyroidism. There was no related basic diseases of respiratory and cardiovascular system in the past. She failed to be weaned from invasive ventilation multiple times after routine treatment and was finally successfully weaned on day five of receiving the combination therapy of a high-dose methylprednisolone intravenous drip and levothyroxine oral administration.

**Conclusion:** This case report indicates that hypothyroidism may be a cause of type II respiratory failure, heart failure, and massive pericardial effusion without cardiac tamponade and that a combination of levothyroxine and corticosteroids could effectively treat the disease. Clinical workers should consider the role of thyroid function in diagnosis, and the admission team should include this aspect in the monitoring scope. Moreover, the role of hormones in the treatment of patients with severe hypothyroidism should not be ignored, and timely treatment should be provided.

## Introduction

Hypothyroidism is defined as insufficient secretion or inadequate function of thyroid hormone in the target tissue. It is commonly observed in outpatient settings and seldom requires hospitalization. However, rarely, the disease can affect the metabolism of multiple organ systems and lead to dysfunction; such cases are primarily divided into two categories as follows: 1) primary hypothyroidism; 2) secondary hypothyroidism, e.g., central thyrotropin-releasing hormone or thyroid-stimulating hormone deficiency, or consumptive hypothyroidism caused by excessive thyroid hormone inactivation. In most patients, the oral administration of exogenous synthetic thyroid hormone can significantly relieve the disease (Almandoz and Gharib, 2012). The current case report describes the case of an older woman with heart failure, type II respiratory failure, and massive pericardial effusion caused by hypothyroidism in a cardiac intensive care unit (CICU). It discusses her clinical manifestations, diagnosis, and treatment, shares experiences related to her diagnosis, treatment, and weaning difficulties, and presents conclusions on glucocorticoids therapy, based on the results.

## Case presentation

The patient was a 64-year-old woman who had intermittent dyspnea with edema in both lower limbs for 15 days. Prior to this, she had been physically healthy, and she had no family history of related genetic diseases and no recorded history of smoking or alcohol consumption. Her mother died of a heart attack. The patient’s physical examination results were as follows: temperature, 36.5°C; pulse, 66 beats/min (bpm); respiration, 18 times/min; blood pressure (BP), 95/63 mmHg. Both lungs had a thick breathing sound (recorded *via* auscultation). Dry and wet rales could be heard at the bottom of both lungs, and there was moderate edema in both of the lower extremities. The primary laboratory data at the time of admission are shown in Table 1.

**TABLE 1:** The patient’s laboratory data.

Parameter	Values	References value	Unit
PH	7.22	7.35–7.45	-
Lac	0.6	0.5–2.2	mmol/L
HCO3−	43.0	18.0–23.0	mmol/L
BE	15.3	−2.0–3.0	mmol/L
PCO2	105	35–48	mmHg
PO2	46	83–108	mmHg
WBC	10.02	3.5–9.5	10∧9/L
NE	0.86	0.40–0.75	%
NE#	8.57	1.80–6.30	10∧9/L
Hb	127	115–150	g/L
PLT	209	125–350	10∧9/L
CK	1050	40–200	U/L
CKMB	36.5	0.0–25.0	U/L
Mb	140	0–107	ng/ml
Tn	<0.05	0–0.05	ng/ml
BNP	361	0–100	pg/ml
D-dimer	348	100–600	ng/ml
α-HBDH	306	72–182	U/L
LDH	434	120–250	U/L
PCT	0.27	0.00–0.50	ng/ml
TSH	11.078	0.35–4.94	uIU/ml
FT3	<1.64	2.43–6.01	pmol/L
FT4	<5.15	9.01–19.05	pmol/L
A-TPO	791.9	0–5.61	IU/ml
A-Tg	>1000.00	0–4.11	IU/ml
E2	39.60	<59.45–241	pmol/L
P4	0.84	<0.54–1.08	nmol/L
PRL	183.10	117.68–505.7	mIU/L
FSH	30.63	11.9–108.7	mIU/ml
TE	<0.17	<0.56–1.77	nmol/L
LH	7.58	15.4–53.3	mIU/ml
Cor	472.92	<276.0	nmol/L
AST	35.0	13.0–35.0	U/L
ALT	15.2	7.0–40.0	U/L
TB	9.3	0.0–21.0	umol/L
DBIL	1.8	0.0–6.8	umol/L
IBIL	7.5	5.0–20.0	umol/L
BUN	5.18	3.1–8.8	mmol/L
sCr	58.3	41–81	umol/L
UA	401	150–360	umol/L
hs-CRP	15.63	0–3.5	mg/L
ACTH	6.75	1.6–13.9	pmol/L
TC	6.13	2.6–6.0	mmol/L
TG	1.25	0.28–1.80	mmol/L
HDL	2.07	0.76–2.10	mmol/L
LDL	3.29	2.06–3.10	mmol/L
AMA- M2	110.1	0–20	units

Lac, lactic acid; BE, base excess; WBC, white blood cell; NE, neutrophils; PLT, platelets; Hb, hemoglobin; CKMB, creatine kinase MB; Mb, myoglobin; Tn, troponin; BNP, brain natriuretic peptide; α-HBDH, alpha-hydroxybutyrate dehydrogenase; LDH, lactate dehydrogenase; PCT, procalcitonin; TSH, thyroid-stimulating hormone; FT3, free triiodothyronine; FT4, free thyroxine; A-TPO, thyroid peroxidase antibody; A-Tg, thyroglobulin antibodies; E2, estradiol; P4, progesterone; PRL, prolactin; FSH, follicle-stimulating hormone; TE, testosterone; LH, luteinizing hormone; Cor, cortisol; AST, aspartate aminotransferase; ALT, alanine transaminase; TB, total bilirubin; DBIL, direct bilirubin; IBIL, indirect bilirubin; BUN, blood urea nitrogen; sCr, serum creatinine; UA, uric acid; hs-CRP, high-sensitivity C-reactive protein; ACTH, adrenocorticotropic hormone; TC, total cholesterol; TG, triglyceride; LDL, low-density lipoprotein; HDL, high-density lipoprotein; AMA-M2, anti-mitochondrial M2 antibody.

**FIGURE 1:**
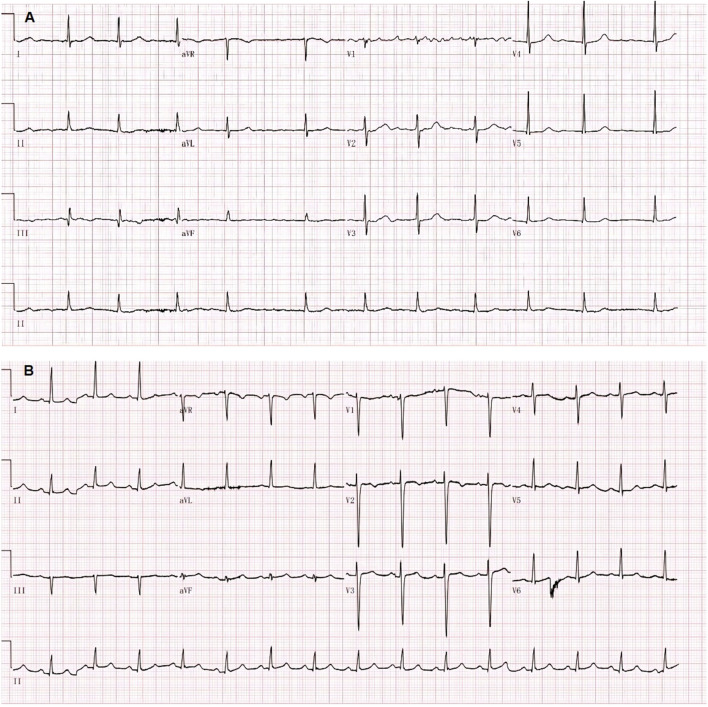
Changes in the electrocardiogram. **(A)** after admission; **(B)** on day twelve of admission.

Electrocardiogram (ECG) showed a low T-wave that was flat and inverted in leads II and III, and aVF (Figure 1). Ultrasonic cardiography (UCG) showed massive pericardial effusion, left ventricular wall thickening, segmental wall motion abnormality, decreased left ventricular systolic and diastolic function, and a mild increase in pulmonary pressure in the mitral, tricuspid, and aortic valves. Bedside colour-Doppler ultrasonography indicated bilateral pleural effusion, with right-side pleural effusion being more obvious. The patient was given a primary diagnosis of “heart failure, massive pericardial effusion, type II respiratory failure, and hypothyroidism,” admitted to the hospital, and given invasive ventilator auxiliary support treatment, and performed by the right thoracentesis and pericardiocentesis. Diuretics were delivered intravenously. According to the consultation opinion of the endocrinology department, the patient was given levothyroxine 25 ug orally once daily (QD).

On the fourth day after admission, the patient’s ventilator parameters were low, which implied that she may no longer require invasive ventilation. She could breathe autonomously following adjustment to continuous positive airway pressure mode. Twenty minutes after the initial weaning test, the patient experienced dyspnea, decreased blood oxygen saturation, elevated BP, an accelerated heart rate (HR), and failed to wean. On day seven after admission, at 8:00, the patient began an attempt to wean. After a 1-h weaning test, the patient had no obvious discomfort, and the tracheal intubation was removed. At 12:00 midday, her BP was 244/100 mmHg, her heart rate was 100 bpm, and she had dyspnea. The patient was given a dexamethasone sodium phosphate injection (10 mg, intravenous push), lobelin (9 mg, intravenous push), nikethamide (1.125 g, intravenous push), and isosorbide dinitrate (30 mg, intravenous pump at 5 ml/h). At 12:30, the patient was vaguely conscious and still had dyspnea. An urgent blood gas analysis showed that the carbon dioxide partial pressure could not be measured. Thus, the anesthesiology department was contacted for emergency endotracheal intubation and this patient was given invasive ventilator ventilator auxiliary support treatment. At 12:40, the patient’s BP was 140/60 mmHg, and her HR was 68 bpm; any changes in her condition were closely observed. According to the consultation opinion of the chief physician of the endocrinology department, methylprednisolone (80 mg, QD) was given on the same day. On day three of methylprednisolone treatment (day nine after admission), the patient had dyspnea and sweating 10 min after the weaning test and had to maintain the original ventilator mode and parameters. On day five of methylprednisolone treatment (day 11 after admission), the patient underwent a weaning test at 9:50, and her vital signs were stable. At 11:00, she was given non-invasive ventilator-assisted respiration. On day 12 of admission and day 6 of methylprednisolone treatment, the oxygen concentration of the non-invasive ventilator was reduced to 40%, and the patient evidenced no obvious discomfort. One hour later, the non-invasive ventilator-assisted ventilation was stopped, and the patient changed to using a mask for oxygen inhalation. By day seven of methylprednisolone treatment (day 19 after admission), the patient did not complain of any obvious discomfort, and the methylprednisolone was stopped. The patient then gradually changed from using a mask to wearing a nasal catheter, and finally, to normal breathing.

The overall treatment protocol was as follows. This patient began taking levothyroxine orally (25 ug, QD) after admission. On day six of admission, the dose was adjusted to 50 ug QD; on day seven following admission, the dose was adjusted to 100 ug, QD; on day 10, another adjustment was made to 125 ug, QD. Thereafter, the dosage was taken orally (125 ug, QD). Once discharged, the patient’s dose was regularly adjusted in the endocrine clinic. On day seven of admission, methylprednisolone (80 mg, QD) was administered for a total of 7 days.

**FIGURE 2:**
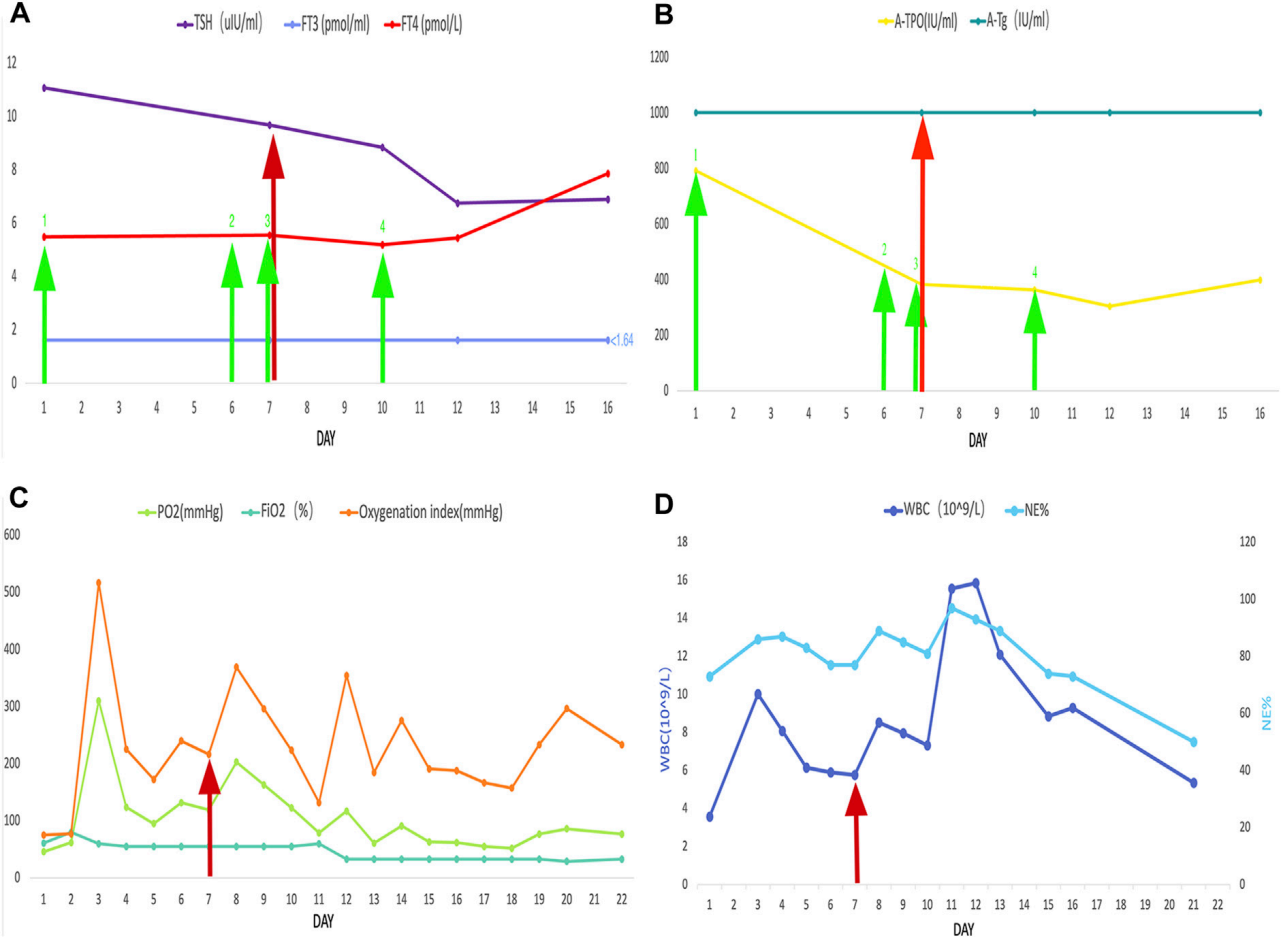
The red arrow represents the start of the use of glucocorticoids. The green arrow represents the levothyroxine administration. The numbered green arrows indicate the administered doses as follows: 1, 25 mg/d; 2, 50 mg/d; 3, 100 mg/d; 4, 125 mg/d. The following are also shown: **(A)** thyroid-stimulating hormone, free triiodothyronine, and, free thyroxine; **(B)** thyroid peroxidase antibody and thyroglobulin antibodies; **(C)** PO_2_, FiO_2_, and oxygenation index; **(D)** white blood cell count and NE%.

**FIGURE 3:**
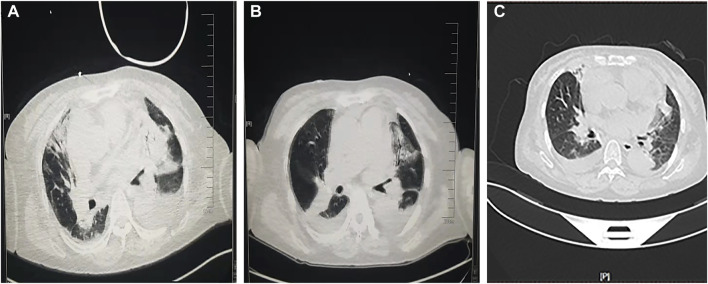
A comparison of pulmonary computed tomography **(A)** before glucocorticoids administration, **(B)** after weaning, and **(C)** before discharge.

The ECG changes before and after the treatment are shown in Figure 1. The dynamic UCG evolution is shown in Table 2 and illustrates that EF increased from 41% to 54% after prompt and effective pericardial and thoracic puncture, continuous pericardial and thoracic drainage, and following the application of diuretics, glucocorticoids, thyroid hormone, and myocardial nutrition drugs. As a result, the patient’s thyroid functioning changed. The fraction of inspired oxygen improvement, as well as white blood cell count throughout the therapy process are shown in Figure 2. The CT changes in the patient’s lungs are shown in Figure 3.

**TABLE 2:** The dynamic evolution of UCG.

Testing time	AO (mm)	LAD (mm)	LVEDD (mm)	LVPW (mm)	RVD (mm)	CO (L/min)	LVEF (%)	SV (ml)	E/A	Diastolic fluid level width of pericardial effusion
On admission	32	34	44	11	24	3.5	41	46	51/72 (0.71)	Posterior wall of left ventricle 22 mm
D5 after admission	34	36	48	11	26	2.7	50	47	74/47 (1.57)	Posterior wall of left ventricle 3 mm
D20 after admission	34	36	49	11	24	4.8	54	60	72/92 (0.78)	Posterior wall of left ventricle 3 mm

AO, aorta; LAD, left atrial anteroposterior diameter; LVEDD, left ventricular end diastolic diameter; LVPW, left ventricular posterior wall; RVD, right ventricular anteroposterior diameter; CO: cardiac output; LVEF, left ventricular ejection fraction; SV, stroke volume; E/A: ratio of E to A.

Five months after being discharged from the hospital, the patient received a follow-up telephone call and noted no particular discomfort.

## Discussion and review of the literature

Thyroid hormone affects the functioning of nearly all of the body’s organs. Hypothyroidism can lead to metabolic disorders and organ diseases but generally does not require hospitalization. The case reported herein concerned severe hypothyroidism with heart failure, type II respiratory failure, and severe pericardial effusion, which are all critical conditions. The patient had to be admitted to the CICU for invasive ventilation, to receive high doses of steroid hormone, standard doses of thyroid hormone, and other treatments.

### The effects of thyroid hormone deficiency on the body’s organs

The authors conducted a literature search and found that kidney damage and hypothyroidism could impact one another (Liakopoulos et al., 2009; Montasser et al., 2010). Respiratory failure (Finsterer et al., 2002; Fukusumi et al., 2014), heart disease (Udovcic et al., 2017; Rastogi et al., 2018), facial paralysis and pituitary hyperplasia (Han et al., 2012; Sharma et al., 2020), as well as myopathy (Lin et al., 2020), can also be caused by hypothyroidism. The clinical features of cases of severe hypothyroidism are summarized in Table 3. Currently, there are few reports on simultaneous respiratory failure, heart failure, and acute disease.

**TABLE 3:** Clinical features of cases regarding severe hypothyroidism.

Case (ref.)	Age (year)/Sex	Initial presentation	Physical examination	Involved organ	Treatment	Prognosis
1) Liakopoulos et al. (2009)	28/F	·constipation	·paleness of skin	·acute renal failure	·thyroid hormone substitution	after 2 years of follow-up: renal function remains normal
			·nonpitting edema of lower extremi-ties			
			·no goiter			
2) Fukusumi et al. (2014)	70/M	·easy fatigability	·GCS: 6	·respiratory failure	·oral levothyroxine	6 years later: continuously dependent on NIPPV
			·no goiter or thyroid bruit	·bilateral pleural effusions	·under steroid cover		
			·unresponsive	·apnea hypopnea	·mechanical ventilation		
3) Rastogi et al. (2018)	30/F	·decreased appetite	·tachypneic; ·normotensive, and afebrile	·DCM	·thyroid hormone replacement therapy	during the course of treatment, her LV function and clinical symptoms improved	
		·progressive shortness of breath on exertion			·conventional therapy for DCM	
		·swelling all over body for 6 months				
4) Han et al. (2012)	15 patients: average 24/6 M 9F	·acratia, aversion to cold, anorexia, facial edema	·not applicable	·pituitary tumorous hyperplasia	·substitute therapy (L-T4, plasma T3, T4, TSH, PRL)	after 4–11 months of substitute therapy: the enlarged pituitary returned to normal size
		·pretibial myxedema				
		·hypoevolutism				
5) Lin et al. (2020)	65/M	·5-year history of gradually progressive dyspnea and pedal edema	·distant heart sounds	·massive pericardial	·thyroxin replacement therapy	after 3 months follow-up: echocardiography was normal
				effusion with tamponade and an EF		
				of 20%–25%		

F, female; M, male; LV, left ventricle; GSC, glasgow coma scale, NIPPV, noninvasive positive pressure ventilation; DCM, dilated cardiomyopathy; EF, ejection fraction.

### The relationship between respiratory failure, heart failure, and hypothyroidism

Type II respiratory failure, also known as hypercapnic respiratory failure, refers to the inability of the respiratory system to oxygenate and expel carbon dioxide (Phoung and Virginina, 2014). Common pathogenies include airway obstruction, kyphosis, and other restrictive pulmonary ventilation disorders, congenital or traumatic chest-wall deformities (e.g., flail chest), neuromuscular diseases (e.g., phrenic paralysis, myopathy, and muscular dystrophy), and diaphragm disorders (e.g., paralysis and congenital diaphragmatic hernia) (Lin et al., 2020). Hypothyroidism, as a cause of type II respiratory failure, is rarely encountered in clinical practice and can easily be overlooked. According to previous researches, the pathophysiological mechanisms of this condition are as follows: slow metabolism; respiratory muscle weakness; diaphragm dyskinesia; a slow respiratory rate and decreased ventilation function; a decreased driving ability of the respiratory center and decreased response to high carbon dioxide and hypoxic stimulation; edema of the tongue, nasal mucosa, and larynx, which exerts pressure on the respiratory tract, causes sleep apnea syndrome, and aggravates dyspnea and hypoxia. Pulmonary interstitial and alveolar edema decrease pulmonary compliance, ventilation function, and alveolar diffusion function, and cause a pulmonary ventilation/blood-flow-ratio imbalance, which is further complicated by heart disease and infection (Jain et al., 1973; Siafakas et al., 1992; Siafakas and Bouros, 1993; Behnia et al., 2000).

The exact pathogenesis of heart disease caused by hypothyroidism remains unclear. Some studies have shown that hypothyroidism can lead to atherosclerosis by accelerating vascular endothelial dysfunction (Cappola and Ladenson, 2003). This may also be related to the high concentration of low-density lipoprotein cholesterol that results from hypothyroidism (Mayer et al., 2006). Therefore, thyroid hormone should also be monitored by admission teams in the cardiology and respiratory departments to reduce the rate of missed diagnoses.

### Factors impacting weaning

Acidosis and changes in mental state are common reasons to induce mechanical ventilation in patients with severe hypothyroidism (Behnia et al., 2000). Whether a patient can wean primarily depends on three factors, i.e., the improvement of the primary disease, the amount of available sputum and the patient’s ability to cough up sputum, and the patient’s state of consciousness (Shaikh et al., 2014). Existing studies have shown that the condition of patients with hypoxemia and hypercapnia can be effectively improved by treatment with levothyroxine for 7–10 days (Ladenson et al., 1988; Behnia et al., 2000). In the current case, the weaning failed on days four and seven of levothyroxine but was successful on day five of methylprednisolone combined with levothyroxine.

Thyroid hormone plays a crucial role in the body’s water–sodium balance and hemodynamics (Capasso et al., 1999). Traditionally, glucocorticoids can cause fluid and sodium retention and, given this, patients with heart failure should not use these drugs (or use it with caution). Current evidence suggests, however, that the short-term use of corticosteroids in patients with heart failure is safe and can improve loop diuretic resistance (Liu and Liu, 2014). Some animal experiments have shown that glucocorticoids can, specifically, dilate renal blood vessels, regulate the synthesis and release of atrial natriuretic peptides, and up-regulate atrial natriuretic peptide receptors on vascular endothelial cells, thereby realizing strong diuretic and natriuretic effects (Liu et al., 2006). A small clinical study showed that prednisone had a strong diuretic effect on patients with heart failure, and may be able to improve renal function (Liu et al., 2006). However, a larger randomized and double-blind controlled study is still needed for further clarification in this regard.

Glucocorticoids are endogenous adrenocorticoids (Magiakou et al., 2006) that can stimulate the differentiation and functional development of the lungs (Busada and Cidlowski, 2017) and effectively inhibit the activation of the immune and inflammatory systems (Barnes and Adcock, 2003). It is an effective anti-inflammatory drug for the treatment of respiratory diseases (Adcock and Mumby, 2017a). Although the level of serum cortisol in the patient in the current case was higher than normal (primarily the result of disease stress), the authors nonetheless decided to intervene with high-dose glucocorticoid treatment, considering the consultation opinions of the endocrinology department.

In the lungs, the glucocorticoid receptor (GR) plays a crucial role in bronchoconstriction, micro-vasoconstriction, immune surveillance maintenance, and alveoli patency (Barnes, 2004; Adcock and Mumby, 2017b). Leseney et al. (1987) demonstrated that, *in vitro*, low thyroid hormone levels could inhibit the expression of the GR, causing hormone resistance. The increase of the serum thyroid hormone level after thyroid hormone replacement therapy will increase the expression of the GR, enhance the combination of the GR and glucocorticoids, and, finally, enhance the efficacy of glucocorticoids. In the current case, after continuous diuretic treatment, the patient still failed to wean until glucocorticoid administration. The current case report results further supported the therapeutic effect of glucocorticoids combined with thyroid hormone in respiratory failure caused by severe hypothyroidism.

## Conclusion

According to the specific conditions of the patients, clinical workers should consider the role of thyroid function in diagnosis, and the admission team might need to include this aspect in the monitoring scope. For patients with severe hypothyroidism, glucocorticoids treatment may be needed, particularly if weaning difficulties occur. The early application of levothyroxine is a key intervention in the treatment of these patients.

## Data Availability

The original contributions presented in the study are included in the article/supplementary material, further inquiries can be directed to the corresponding author.

## Abbreviations

TRH, thyrotropin-releasing hormone; TSH, thyroid-stimulating hormone; CICU, cardiac intensive care unit; bpm, beats per min; BP, blood pressure; ECG, electrocardiogram; UCG, ultrasonic cardiography; CPAP, continuous positive airway pressure; HR, heart rate; FiO_2_, fraction of inspiration O_2;_ WBC, white blood cell; CT, computed tomography; GC, glucocorticoid; GR, glucocorticoid receptors; QD, once a day, quaque die; AO, aorta; LAD, left atrial anteroposterior diameter; LVEDD, left ventricular end diastolic diameter; LVPW, left ventricular posterior wall; RVD, right ventricular anteroposterior diameter; CO, cardiac output; LVEF, left ventricular ejection fraction, SV, stroke volume; E/A, ratio of E to A.
